# Comparative Analysis of Hydrate Nucleation for Methane and Carbon Dioxide

**DOI:** 10.3390/molecules24061055

**Published:** 2019-03-18

**Authors:** Pranav Thoutam, Sina Rezaei Gomari, Faizan Ahmad, Meez Islam

**Affiliations:** School of Science, Engineering and Design, Teesside University, Tees Valley, Middlesbrough TS1 3BX, UK; S6181724@tees.ac.uk (P.T.); F.Ahmad@tees.ac.uk (F.A.); M.Islam@tees.ac.uk (M.I.)

**Keywords:** hydrate formation, nucleation, gas dissolution, induction time, carbon dioxide, methane

## Abstract

Research in the field of hydrate formation requires more focus upon its modelling to enable the researchers to predict and assess the hydrate formation and its characteristics. The main focus of the study was to analyze the deviations induced in various parameters related to hydrate nucleation caused by the choice of different measuring correlations or methods of their sub-components. To serve this purpose under a range of operational conditions, parameters of hydrate nucleation such as rates of nucleation and crystal growth, critical radius of the nucleus, and theoretical induction time for carbon dioxide and methane were considered in this study. From these measurements, we have quantitatively compared the ease of hydrate formation in CO_2_ and CH_4_ systems in terms of nucleation while analyzing how various correlations for intermediate parameters were affecting the final output. Values of these parameters were produced under the considered bracket of operational conditions and distributed among six cases using both general and guest-gas specific correlations for gas dissolution and fugacity and their combinations. The isotherms and isobars produced from some of the cases differed from each other considerably. The rate of nucleation in one case showed an exponential deviation with a value over 1 × 10^28^ at 5 MPa, while the rest showed values as multiples of 10^6^. These deviations explain how sensitive hydrate formation is to processing variables and their respective correlations, highlighting the importance of understanding the applicability of semi-empirical correlations. An attempt was made to define the induction time from a theoretical perspective and derive a relevant equation from the existing models. This equation was validated and analyzed within these six cases from the experimental observations.

## 1. Introduction

Gas hydrates are solid crystalline compounds generally formed when guest gas molecules are trapped inside the cages formed by water molecules at high pressure and low temperature conditions [1]. Gas hydrates have attracted much recent research attention due to their potential employment for various purposes such as desalination, acidic gas capture, gas storage and safe transportation [2]. Mathematically, the hydrate formation remained unpredictable due to the lack of efficient mathematical models that are applicable for wide ranges of hydrate formation conditions and reactors [3,4]. Apart from apparatus design, there has been extensive research into hydrate formation under the influence of various physical materials such as gels, nano-particles, and foams, chemical additives such as semi-clathrates and thermodynamic additives, various guest gas mixtures and sundry combinations of the above [2]. Over the years, evaluations of the characteristics of hydrate formation through experimental observation have been more frequent than modelling studies, which has hindered the development of hydrate modelling [3]. A few proposals were made in the modelling aspect of hydrate formation, making predictions based on their own sets of customized parameters [5,6,7,8,9,10,11,12,13,14]. Most models contained equations whose components were not presumptive and hence experimental observations were required to calculate the unknown parameters.

Kashchiev and Firoozabadi [9,10,11,15] proposed a theoretical model which explained how to characterize hydrate nucleation through a thermodynamic analysis without the requirement of pertinent experimental derivations. However, their model was limited to a quiescent pure system with a single guest gas, and thus was not widely applicable. In addition, it is incapable of calculating crucial variables such as overall hydrate growth rate, the effect of semi-clathrates or the physical hindrance caused by heterogeneous nucleation at the gas-liquid interface upon bulk homogeneous nucleation. Hence further exploration and analysis was needed to design a mathematical model able to reliably predict the parameters of hydrate formation such as rate of nucleation, induction time and growth rate of hydrate crystal among others. Furthermore, a descriptive understanding of various parameters that could affect hydrate formation needed to be developed in order to produce a viable, integral and self-sufficient mathematical model that could shed light upon the overall process and to generate accurate results.

Due to the involvement of gas dissolution, hydrate nucleation and crystal growth, hydrate formation is a complex process to mathematically simulate, where changes in each stage can affect the final output of successive stages. Hence, the main findings using Kashchiev and Firoozabadi’s model are affected by the concentration of gas in liquid, as well as being affected by the attributes of the system such as phase properties and operational conditions [9,10,11]. Kashchiev and Firoozabadi used Henry’s and the Peng-Robinson (PR) models to calculate the concentration in liquid and fugacity of gas respectively. There have been many models proposed to calculate the dissolved gas concentration which also proposed fugacity under various operational conditions [16,17,18,19,20,21]. However, these models have been superseded by other models that are designed to be gas specific with higher accuracy [22,23,24,25]. In order to understand how important it is to take advantage of these improvements in current modelling, the premises which govern the relevant correlations and how much the latter are influenced by their internal parameters under various operational conditions need to be determined. Each correlation has its own set of parameters representing an attribute of the recipient, contributing to its accuracy and domain of applicability. It is important to understand how these parameters affect the final output so as to check if the associated error falls within a region of tolerance and in which operational conditions the error is acceptable. The calculation and analysis of the characteristics of hydrate formation with guest gases being either CO_2_ or methane using various correlations is conducted to investigate any additional governing factors that enhance the result’s variability.

In comparative quantitative analyses of nucleation parameters, it has become conventional to use the method proposed by Kashchiev and Firoozabadi [9,10,11]. The main concern here is the use of the same underlying correlations, such as Henry’s law for gas dissolution and the PR model for fugacity, in generating the final values of the parameters. This study focuses upon how generalized and gas specific semi-empirical models for gas dissolution would affect the final outputs of Kashchiev model and their applicability. In this study, the values of nucleation parameters are generated in six different ways using the combinations of various gas dissolution and fugacity correlations. These values and their corresponding profiles of deviation will provide an understanding of how the correlations for dissolution and fugacity influence the final output of nucleation parameters such as rate of nucleation, induction time, and hydrate volume fraction. In addition, there is no universal agreement upon the measurement process for induction time. This can lead to uncertainty in its measured values for the hydrate formation experiments despite the similar set of conditions when different techniques are used. This might have been influenced by various available measuring techniques such as gas uptake, temperature fluctuation and temporal evolution of light passed through the reactor to detect progressive nucleation [12,13,14]. Despite the availability of multiple measuring techniques, in order to connect the practical observations to the theory by means of mathematical modelling, a generalized theoretical definition is needed. Due to the interference of various inhibitors depending upon the exothermic nature and location of hydrate formation along with the unavailability and difficulty of gas to form the hydrates, despite the initial nucleation in the system, the process as a whole may not progress into an active and continuous growth state causing some of the detection techniques to be unable to identify the induction time. Especially, the detection would be erratic when using light scattering and temperature raise for nucleation in quiescent systems with a probability of localized hydrate formation with low kinetics. To address these issues by assessing hydrate formation from a theoretical perspective using modelling, a generalized definition and corresponding methods or equations are needed. In this study, equations to calculate theoretical induction time and the volume fraction of hydrate crystals were proposed, which could be further developed for integration into an efficient quantitative assessment system. The practical applicability of this theoretical induction time was validated through experimental analysis. Besides the influence of various measuring correlations and the deviations were discussed. This included an explanation from the perspective of physical properties of the guest gas.

## 2. Methodology

This study mainly focused upon the influence of dissolved gas concentration and fugacity in calculating various hydrate formation parameters such as rate of nucleation, induction time, extent of crystal growth and the volume fraction of hydrates at the theoretical induction time. A quiescent system consisting of pure water and supplied with pure guest gas (CO_2_ or CH_4_) excluding any physical or chemical interventions was considered. CO_2_ and CH_4_ are two gases that have been frequently studied for use in hydrate formation for various applications [26,27,28]. This study mainly focused upon the primary nucleation when the system is free of hydrates and considers the ease of hydrate formation and the sensitivity of the correlations of hydrate parameters and their respective deviations from one another. In order to achieve a reliable comparison between the two gas systems, the properties of methane gas hydrates were kept the same as those given in Kashchiev and Firoozabadi including homogeneity [9,10,11]. However, the superficial energy barrier for CO_2_ hydrate was taken as 14 mJ/m^2^ and the volume of the hydrate building unit for sI hydrate was 0.151 nm^3^ [29]. Two correlations were employed for the calculation of dissolved gas concentration that are derived from Henry’s law and the model proposed by Duan et al., with the latter considered mainly due to its applicability in hydrate formation conditions [22,23,24,25]. Various models are available for the calculation of gas dissolution in the presence of hydrate in the system, which were not considered in this study due to the focus being primary nucleation having no hydrate in the system [30,31,32].

### 2.1. Cases

For ease of comparison and to maintain well-defined boundaries among the final results, the analysis was divided into six cases, with each representing the introduction of a new parameter and/or a new correlation for an existing parameter and/or combinations of both to the previous case. We derived some of the equations using existing correlations or from combinations of multiple correlations to suit the study’s requirements. Further details are given in the following sections.

In the course of the study, the following assumptions were made in accordance with Kashchiev and Firoozabadi [10]:

- Progressive nucleation occurs only after the water becomes saturated with dissolved gas at the given operational conditions.
- Guest gas is evenly distributed throughout the solution.
- Negligible temperature fluctuations exist in the system during the process of primary nucleation.
- Nucleation sites are situated far enough from each other to eliminate mutual interruptions before either of them attains the critical radius.
- Both nucleation and crystal growth occur only through the undisturbed diffusion of dissolved gas.
- Homogeneous nucleation is considered for the modelling (not for the experimental validation) which is not hindered by other factors such as nucleation at the gas-liquid interface.

Homogeneous nucleation is considered for the modelling to enable the results from the following cases comparable to the results given by Kashchiev and Firoozabadi [11]. However, in realty, for having a lesser surface energy barrier, primary nucleation always occurs in the heterogeneous media such as the gas-liquid interface along the wall. This is further discussed in our experimental validation.

#### 2.1.1. Case 1

The first case was designed to have ideal mixing of the guest gas with ideal characteristics. To meet these requirements, the basic Henry’s law equation for solubility was used [16]. The dissolved gas concentration in the liquid phase according to Henry’s law was as follows:

(1) $$X_{i}=\;H_{i}P,$$

where *H_i_* is Henry’s constant for the guest gas ‘*i*’, *P* is the operational pressure and *X_i_* is the dissolved gas mole fraction in liquid. In this study, H_CO2_ = 3.3 × 10^−4^ and H_CH4_ = 1.4 × 10^−5^ [33]. Due to the ideal conditions, the compressibility of the guest gas was ignored and hence the fugacity term was omitted in this case.

#### 2.1.2. Case 2

The fugacity term was introduced into the system, where coefficient of fugacity ($\emptyset_{i})$ values were calculated from Peng-Robinson (PR) equation of state. The corresponding equation was as follows:

(2) $$X_{i}=\;\emptyset_{i}H_{i}P.$$

Since Equation (2) was used in Kashchiev and Firoozabadi’s [9,10,11] model of nucleation, it plays a pivotal role in the current study. This was considered to be the reference case for the entire analysis. We calculated rate of nucleation as guided by the Kashchiev and Firoozabadi model in this case and compared with the profiles given in their study [10].

#### 2.1.3. Case 3

Japas and Sengers (1989) [34] experimentally observed that, for aqueous and non-aqueous solvents, Henry’s law constant follows a parabolic profile where it reaches a maximum value and then tends to fall. This work was continued by Harvey (1996) [35], who proposed an equation for the temperature-dependent dimensionless Henry’s law constant (k_H_) for CO_2_ and CH_4_ through curve fitting to experimental values, which was as follows:

(3) $$\ln\left(k_{H}\right)=\ln\left(p_{l}^{s}\right)+\frac{A_{H}}{T_{r}}+\;\frac{B_{H}}{T_{r}}{\left(1-T_{r}\right)}^{0.355}+\frac{C_{H}}{T_{r}^{0.41}}e^{\left(1-T_{r}\right)},$$

where $p_{l}^{s}$ was the saturation pressure of solvent (water), *T_r_* was the reduced temperature, and *A_H_, B_H_* and *C_H_* were the parameters of the correlation which are shown in Table 1. Equation (3) was substituted into Equation (2) in order to calculate the concentration of dissolved gas.

(4) $$X_{i}=\;\frac{\emptyset_{i}P}{k_{H}}.$$

#### 2.1.4. Case 4

The activity coefficient (γ_i_) shows the electromagnetic interactions among the ions available in a system. In most cases, the activity coefficients of aqueous CO_2_ or CH_4_ are taken to be equal to unity. However, according to Diamond and Akinfiev (2003) [36], the coefficients were observed to deviate from unity at low temperatures. They also mentioned that, at low gas concentrations in the liquid phase, considering the activity coefficient to be unity was expected to produce reliable values of dissolved gas concentration. Since this study investigates how the deviations produced by various correlations had an impact on the final output, dissolved gas concentration was measured using non-unity activity coefficients. The corresponding dissolved gas concentration equation cited by Diamond and Akinfiev was:

(5) $$X_{i}=\;\frac{\emptyset_{i}P}{k_{H}\gamma_{i}}.$$

Various models were available proposing different correlations to measure the activity coefficients of different gases under given operational conditions [36,37,38,39]. However, coefficients for these correlations were not widely available for methane, and hence the activity coefficient equation was derived from the gas dissolution model proposed by Duan et al. (1992) [22].

The chemical potential of any system was defined as its ability to provide molecules to surrounding systems. In proposing his equation of state for the H_2_O-CO_2_-CH_4_ system, Duan et al. (1992) [22] used the following equations to measure chemical potential in both the vapor and liquid phases:

(6) $$\mu_{i}^{v}=\;\mu_{i}^{v}{}^{0}+RT\;lnf_{i},$$

(7) $$\mu_{i}^{l}=\;\mu_{i}^{l}{}^{0}+RT\;lna_{i},$$

where $\mu_{i}^{l}$ and $\mu_{i}^{v}$ were the chemical potential of the guest gas under the operational conditions in dissolved and vapor state respectively, $\mu_{i}^{l}{}^{0}$ and $\mu_{i}^{v}{}^{0}$ were the initial chemical potentials of the guest gas in dissolved state and vapor state respectively, *R* was the gas constant, *f_i_* and *a_i_* were the fugacity and activities of respective guest gas ‘*i*’. Under the phase equilibrium conditions, the chemical potential of each component in one phase was equal to the chemical potential in the other phase. From Equations (6) and (7), the following equation was deduced:

(8) $$\gamma_{i}=\;\frac{\emptyset_{i}P}{m_{i}}e^{\left(\frac{\mu_{i}^{l}{}^{0}-\;\mu_{i}^{v}{}^{0}}{RT}\right)},$$

where *m_i_* was the molality of the dissolved gas. As $\mu_{i}^{l}{}^{0}$ represents the chemical potential of an ideal solution, while $\mu_{i}^{v}{}^{0}$ represented the ideal gas, the main focus here was on their difference rather their absolute values and thus $\mu_{i}^{v}{}^{0}$ was set to zero [40].

(9) $$\gamma_{i}=\;\frac{\emptyset_{i}P}{m_{i}}e^{\left(\frac{\mu_{i}^{l}{}^{0}}{RT}\right)}.$$

The molality (*m_i_*) was calculated from Duan’s model for gas dissolution, and the activity coefficient calculated from Equation (9) was substituted in Equation (5) to calculate the dissolved gas concentration.

#### 2.1.5. Case 5

The dissolved gas concentration was calculated using a gas specific semi-empirical model proposed by Duan et al. [24,25] specifically for CO_2_ and CH_4_ systems. The model was based on specific particle interaction theory for the liquid phase combined with Duan et al. [22] equation of state. For the dissolution of gas in pure water, the main equation was as follows:

(10) $$\ln\left(m_{i}\right)=\ln\left(y_{i}\emptyset_{i}P\right)-\;\frac{\mu_{i}^{l}{}^{0}}{RT},$$

where y_i_ was the vapor phase mole fraction of the guest gas which, assuming relatively negligible water vapor in the system under the hydrate formation conditions, was set to 1. The coefficient of fugacity term in Equation (10) was measured using the correlation proposed by Duan et al. [22,24].

#### 2.1.6. Case 6

In this case the focal parameter was the coefficient of fugacity. So far, in all the cases, to calculate the rate of nucleation as proposed by Kashchiev and Firoozabadi [10], we used the PR model. For the current case, the driving force term used the coefficient of fugacity correlation proposed by Duan et al. [22,24,25], where two individual correlations correspond to the behavior of CO_2_ and CH_4_ under the operational conditions.

(11) $$\emptyset_{CO_{2}}=\;C_{1}+\left(C_{2}+\;C_{3}T+\frac{C_{4}}{T}+\;\frac{C_{5}}{\left(T-150\right)}\right)P+\left(C_{6}+\;C_{7}T+\frac{C_{8}}{T}\;\right)P^{2}+\left(\;C_{9}+\;C_{10}T+\frac{C_{11}}{T}\right)\;lnP+\frac{\left(C_{12}+\frac{C_{13}}{T}\right)}{P}+\frac{C_{14}}{T}+C_{15}T^{2},$$

(12) $$ln\emptyset_{CH_{4}}=Z-\;1-lnZ+\;\frac{\left(C_{1}+\;\frac{C_{2}}{T_{r}^{2}}+\;\frac{C_{3}}{T_{r}^{3}}\right)}{V_{r}}+\;\frac{\left(C_{4}+\;\frac{C_{5}}{T_{r}^{2}}+\;\frac{C_{6}}{T_{r}^{3}}\right)}{2V_{r}{}^{2}}+\;\frac{\left(C_{7}+\;\frac{C_{8}}{T_{r}^{2}}+\;\frac{C_{9}}{T_{r}^{3}}\right)}{4V_{r}{}^{4}}+\;\frac{\left(C_{10}+\;\frac{C_{11}}{T_{r}^{2}}+\;\frac{C_{12}}{T_{r}^{3}}\right)}{5V_{r}{}^{5}}+\;\frac{C_{13}}{2C_{15}}\;\left(C_{14}+1-\left(C_{14}+1\;+\;\frac{C_{15}}{V_{r}^{2}}\right)e^{\left(-\frac{C_{15}}{V_{r}^{2}}\right)}\right),$$

where *Z* was the compressibility factor, *V_r_* and *T_r_* were, respectively, the reduced volume and temperature of the guest gas, and *c_i_* represents the parameters of the correlation which were shown in Table 2. In addition to these cases, certain terms were defined to ease the description in the analysis, a few modifications were made to the correlations proposed by Kashchiev and Firoozabadi [9,10,11], and several equations were derived from the existing correlations, which are explained in Section 2.1, Section 2.5 and Section 2.6.

### 2.2. Isothermal and Isobaric Processes

There are generally two stances of interpreting the description when using the terms isothermal and isobaric, depending upon the method of usage: (1) Physical conditions for an undertaken process; or (2) method of analyzing the parameters obtained under other conditions. In this study, hydrate formation under various operational conditions was assumed to be both isothermal and isobaric. We used these terms and their analogues to show how the process variables behave when discrete isothermal-isobaric hydrate formation processes were analyzed along the lines of constant pressure or constant temperature. Hence, here the isothermal process was a comparative analysis of parameters obtained from an ensemble of selected hydrate formation processes that were carried out at constant operational temperature, and the isobaric process was a comparative analysis of parameters obtained from an ensemble of selected hydrate formation processes that were carried out at a constant operational pressure. For the process being exothermic, hydrate formation as a whole is not an isothermal process. However, as the current study focuses merely upon the primary nucleation, where there was no prior hydrate formation, we have disregarded the possibility of any temperature fluctuation within the system.

### 2.3. Deviation

Parameters of hydrate formation were measured under various operational pressures (reference pressure = 30 MPa) and operational temperature (reference temperature = 273.2 K) conditions within the limits of conditions favorable for hydration. Their respective deviations from the reference case (Case 2) were plotted against pressure and temperature for both CO_2_ and CH_4_, which were obtained from the following expression:

(13) $$\Delta =\;\left(\frac{\Lambda_{j}-\;\Lambda_{2}}{\Lambda_{2}}\right),$$

while $\Lambda_{j}$ represents the parameter concerned under the given operational conditions calculated for case *j*. If the deviation is higher in any case or at any operational condition, it means the effect of the respective parameter that is causing the deviation is intense. Any changes in the selected model that is used to calculate this parameter could bring a significant change in the final output.

### 2.4. Rate of Nucleation

The model proposed by Kashchiev and Firoozabadi [10] was used to calculate the rate of nucleation under the given conditions with a slight modification in the rate equation which was described below. The following rate equation proposed by Kashchiev and Firoozabadi [10]:

(14) $$J=zf_{e}^{\;*}{\;C}_{0}e^{\frac{\Delta \mu }{\mathrm{kT}}}e^{\frac{-W}{\mathrm{kT}}},$$

where z is the Zeldovich factor, fe* was the frequency of attachment at hydrate equilibrium, C_0_ was the concentration of nucleation sites, Δμ was the super-saturation, w was the work done for successful nucleus formation. The correlations for each of these parameters were provided by Kashchiev and Firoozabadi [9,10].

(15) $$f_{e}^{*}=\;\varepsilon {\left(4\pi c\right)}^{\frac{1}{2}}v_{h}^{\frac{1}{3}}DM_{i0}n^{*\frac{1}{3}},$$

where ε was the sticking coefficient, *c* was the shape factor, *v_h_* was the volume of hydrate building unit, *D* was the diffusivity of dissolved gas in aqueous solution and n* was the number of building units constituting a successful nucleus. Substituting Equation (15) into Equation (14) gives the following equation:

(16) $$J=z\left(\varepsilon {\left(4\pi c\right)}^{\frac{1}{2}}v_{h}^{\frac{1}{3}}DM_{i0}n^{*\frac{1}{3}}\right)C_{0}e^{\frac{\Delta \mu }{\mathrm{kT}}}e^{\frac{-W}{\mathrm{kT}}}.$$

In Equation (16), $M_{i0}$ was defined as the dissolved gas concentration (m^−3^) under hydrate equilibrium conditions, while $e^{\frac{\Delta \mu }{\mathrm{kT}}}$ represented the extrapolation of dissolved gas concentration into the operational conditions. The value of $M_{i0}$ was calculated from Henry’s model, and in this study, $M_{i0}e^{\frac{\Delta \mu }{kT}}$ is termed *M*_i_, which represents the dissolved gas concentration under operational conditions. This reduced Equation (14) into Equation (17):

(17) $$J=z\left(\varepsilon {\left(4\pi c\right)}^{\frac{1}{2}}v_{h}^{\frac{1}{3}}DM_{i}n^{*\frac{1}{3}}\right)C_{0}e^{\frac{-W}{\mathrm{kT}}}.$$

**Figure 1:**
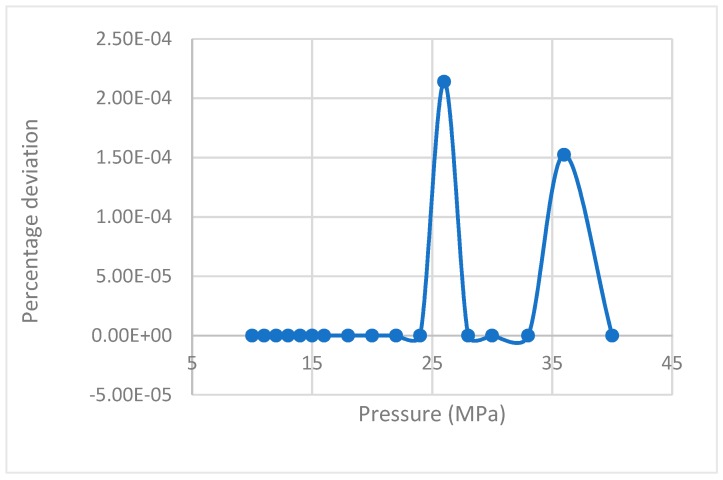
Deviation of rate of nucleation values calculated by Equation (17) from the equation proposed by Kashchiev and Firoozabadi [10].

The values of rate of nucleation for the CH_4_ guest gas for varying pressures were generated using Equations (14) and (17) and their deviations were plotted against pressure, resulting in negligible deviation (Figure 1).

Kashchiev and Firoozabadi [9] defined the work done for the formation of a critical nucleus using Equation (18):

(18) $$W=\frac{8c^{3}v_{h}^{2}\sigma_{ef}^{3}}{27\;\Delta \mu^{2}}.$$

In Equation (18), *σ_ef_* is the effective specific surface energy (J/m^2^) of the hydrate nucleus. From Equations (17) and (18), the following observation was made:

$$J\;\propto \;e^{\left(-\frac{1}{\Delta \mu^{2}}\right)}$$

This could contribute an exaggerated reflection into the rate of nucleation even with a small change in super-saturation (Δμ). Super-saturation was defined using Equation (19):

(19) $$\Delta \mu =kT\ln\left(\frac{\emptyset P}{\emptyset_{e}P_{e}}\right)+\left(n_{w}v_{w}-v_{h}\right)\left(P-P_{e}\right)$$

where *∅_e_* and *P_e_* were, respectively, the coefficient of fugacity and pressure at hydrate equilibrium conditions, *V_w_* was the volume of a water molecule and *n_w_* was the number of water molecules constituting the hydrate building unit.

### 2.5. Induction Time

There had been multiple definitions and methods of experimental measurement for induction time [3,11,13]. In this study, a correlation for theoretical induction time was derived on the basis of Sloan and Koh’s (1998) [13] definition, which was the time elapsed for hydrate formation to become spontaneous. This was governed by the crystal size crossing the critical radius in order to overcome the resistance provided by superficial energy.

In order to derive the theoretical induction time, the equation for the extent of growth at any given time proposed by Kashchiev and Firoozabadi (2003) [11] was equated with the critical radius equation proposed by Sloan and Koh (1998) [13]:

(20) $$r\left(t\right)={\left(Gt\right)}^{m}$$

where *G* was the growth constant and the value of *m* was equal to 0.5 for growth by the undisturbed diffusion of dissolved gas in the formation of spherical crystallites. *G* was measured using Equation (21):

(21) $$G\;=\;2\varepsilon vhDM_{i}\left(e^{\left(\frac{\Delta \mu }{kT}\right)}\;-1\right),$$

(22) $$R_{c}=\;\frac{2\sigma }{\left(\Delta g\right)}$$

where *R_c_* was the critical radius of the hydrate nucleus and Δ*g* was the Gibbs free energy per unit volume of hydrate formation. According to Sloan and Koh (1998) [13], the concerned equation was calculated using Equation (23):

(23) $$-\Delta g=\frac{kT}{v_{h}}\ln\left(\frac{\emptyset P}{\emptyset_{e}P_{e}}\right)+\;\frac{n_{w}v_{w}\left(P-P_{e}\right)}{v_{h}}.$$

Finally, the theoretical induction time ($t_{ind}$) was given using Equation (24):

(24) $$t_{ind}=\frac{\sigma^{2}}{2\varepsilon v_{h}D(M_{i}-M_{i0}{)(\Delta g)}^{2}}.$$

### 2.6. Hydrate Volume Fraction at the Theoretical Induction Time

Kashchiev and Firoozabadi (2003) [11] defined induction time from an experimental perspective on the basis of the temporal evolution of the intensity of light due to crystallization passing through the reactor. They defined induction time as the time taken for the volume fraction of hydrates in the reactor to reach a specific value. An exemplary value of 0.01 of volume fraction was taken in order to produce an integral numerical value for the induction time using Equation (25).

(25) $$t_{ind}^{K}={\left(\frac{3\alpha v_{w}\left(1+3m\right)}{4\pi zf_{e}^{*}{\left(2\varepsilon v_{h}DC_{e}\right)}^{3m}}\right)}^{\left(\frac{1}{1+3m}\right)}\left(e^{\left(\frac{-\Delta \mu }{kT}\right)}{\left(1-e^{\left(\frac{-\Delta \mu }{kT}\right)}\right)}^{\left(\frac{-3m}{1+3m}\right)}\right)\left(e^{\left(\frac{W}{\left(1+3m\right)kT}\right)}\right),$$

where α was the volume fraction of hydrates in the reactor. The main aim of calculating hydrate volume fraction at the end of theoretical induction time was to investigate the validity of this assumption by checking if the values are the same for all the considered operational conditions.

Equation (26) was produced by equating Equations (24) and (25), along with a few adjustments to suit the cases as explained in Section 2.4.

(26) $$\alpha =\left(\frac{4\pi zf_{e}^{*}{\left(2\varepsilon v_{h}DC_{e}\left(M_{i}-M_{i0}\right)\right)}^{3m}}{3v_{w}\left(1+3m\right)}\right)\;{\left(t_{ind}e^{\left(\frac{-W}{\left(1+3m\right)kT}\right)}\right)}^{\left(1+3m\right)}.$$

## 3. Experimental Setup

**Figure 2:**
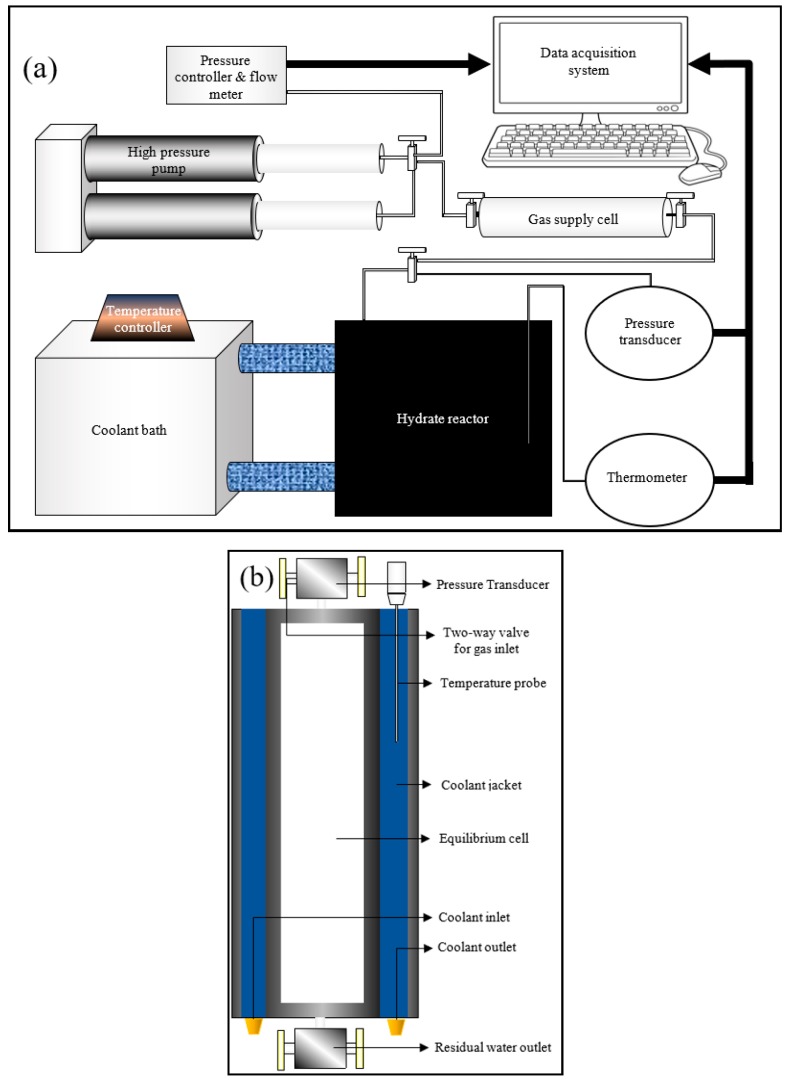
Experimental setup configuration (**a**) and the reactor configuration (**b**).

Even though techniques measuring the kinetics of hydrate nucleation were discussed in the literature, they may not be efficient enough to evaluate the rate of primary hydrate nucleation rate. In order to assess the primary nucleation, finding out the location of primary nucleation is required, which is unpredictable. However, for induction time being an observable quantity, an attempt can be made to bridge the modelling results with the practical results through experimentation by comparing the induction times. The experiments were conducted at hydrate lab facilities of Heriot-Watt University, Edinburgh. A cylindrical jacketed type rocking cell reactor was chosen for the hydrate formation, whose axis was maintained horizontally to promote gas dissolution by increasing the gas-liquid interfacial area. A Quizix high-pressure syringe pump was used to pressurize the gas supply chamber along with coolant circulation through the reactor jacket to maintain the system at isobaric and isothermal conditions. Experimental data such as temperature (K), pressure (psi) and volumetric gas consumption from the gas supply to the reactor (mL) were measured and saved in the data acquisition system. The experimental setup including the overall configuration and the reactor configuration are shown in Figure 2a,b.

A pressure of 3.5 and 12 MPa was chosen for CO_2_ and CH_4_ hydrate formations respectively, while temperature being the same for both the cases at 274.15 K. The reactor was cleaned and vacuumed before starting the experiment to ensure no gaseous contamination in the chamber. Initially, the reactor was maintained at above hydrate formation temperature until the gas consumption becomes stable, indicating the equilibrium gas dissolution under the applied P-T conditions. Then, the temperature was set to the hydrate formation conditions (274.15 K) by initializing the data collection through the acquisition system.

## 4. Results and Discussions

### 4.1. Dissolved Gas Concentration in Aqueous Medium

According to the model proposed by Kashchiev and Firoozabadi [10], the extent of gas dissolution was described as the number of molecules of guest gas dispersed in a unit volume of water, instead of using molarity and molality conventions. To fit the model, the values of dissolution calculated from the aforementioned six cases were converted accordingly.

**Figure 3:**
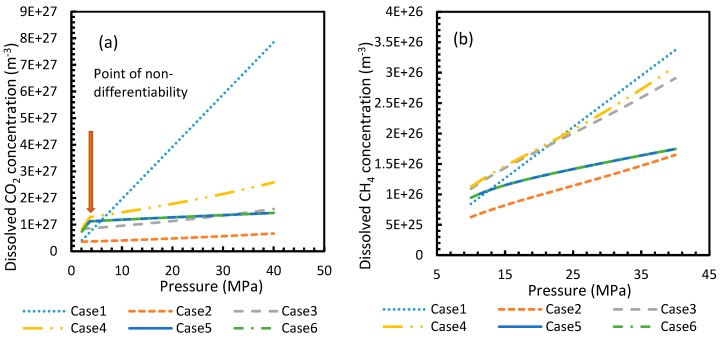
Profiles of dissolved gas concentration for CO_2_ (**a**) and CH_4_ (**b**) in aqueous solution under isothermal conditions at reference temperature 273.2 K. Points of non-differentiability can be viewed in Cases 4, 5 and 6; which are absent in Cases 1, 2 and 3. Values of Case 5 and 6 overlapped with each other.

**Figure 4:**
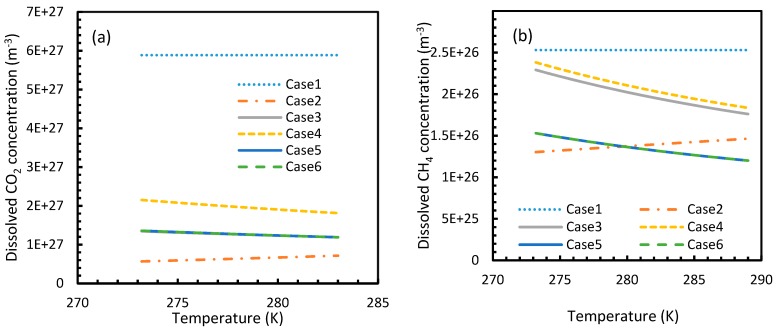
Concentration profiles of dissolved CO_2_ (**a**) and CH_4_ (**b**) in aqueous phase under isobaric conditions at a reference pressure 30 MPa. The ideal case (Case 1) showed no change in its profile due to the lack of the temperature dependent parameter in its dissolution equation. Similar to Figure 1, the values of Case 5 overlapped with Case 6.

**Figure 5:**
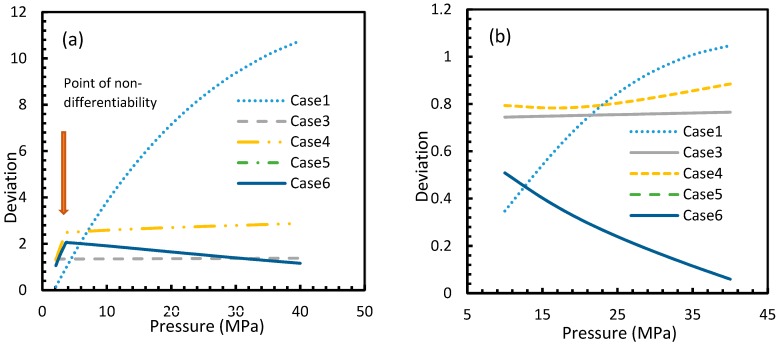
Deviations of gas concentrations of CO_2_ (**a**) and CH_4_ (**b**), calculated through considered cases from the reference case (Case 2). One can observe that the point of non-differentiability was sharply pronounced by these profiles. The overall deviation of Case 4 from Case 3 is negligible within the domain of operational conditions when compared to the rest. Case 1 showed an exponential increase in deviation from Case 2 with pressure, while the rest showed either asymptotic with less magnitude or decremental deviations with pressure from Case 2.

**Figure 6:**
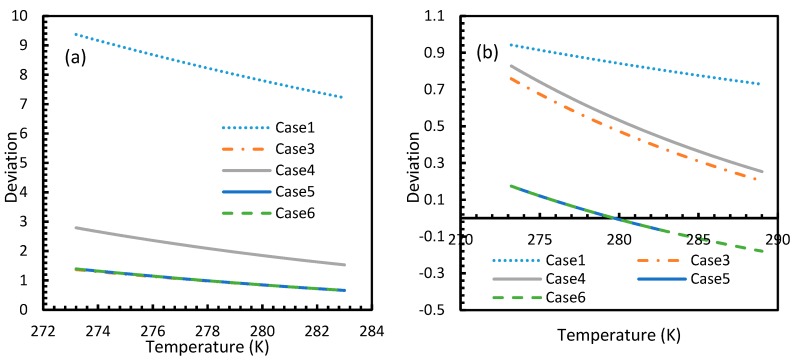
Profiles of deviations of dissolved CO_2_ (**a**) and CH_4_ (**b**) concentrations in aqueous phase calculated under the considered cases from the reference case (Case 2). The depletion in deviations is observed at high operational temperatures. One can see the deviations are considerably high with CO_2_ as the guest gas, compared to CH_4_.

Figure 3 and Figure 4 show that the dissolved gas concentration of CO_2_ is approximately 10 times higher than that of CH_4_ in all cases, making CO_2_ approximately 10 times more available than methane for hydrate formation. From Figure 5 and Figure 6, it was noted that Case 1 was the only one that showed an exponential deviation from Case 2 for dissolved gas concentration with pressure while the rest showed either an asymptotic profile with significantly lower deviations compared to Case 1 or the decreasing profiles. For methane under the isobaric conditions, on the other hand, the values derived from Cases 5 and 6 showed tendencies towards negative deviations at high temperatures. It was also observed that all cases showed positive deviations from Case 2, indicating that Case 2 delivered the lowest values except at high operational temperatures. The deviations were higher in the case of CO_2_ when compared to CH_4_. Hence, at conditions of low pressure and/or high temperature, more accurate correlations are needed in order to obtain relatively acceptable results for the dissolution of gas.

For all of the cases except Cases 1 and 2, in the case of CO_2_ under isothermal conditions, a point of non-differentiability was observed. This point lay where the curve had more than one tangent, and typically two. This point of non-differentiability was observed at 3.5 MPa, which was the bubble point pressure of CO_2_. This point of non-differentiability signifies the behavioral change of the gas at that operational condition. A comprehensive discussion of the effect of bubble point pressure on the final output is provided in Section 4.2. The values of dissolved gas concentration from Cases 5 and 6 mostly coincided with those of Case 3, whereas Case 4 showed an overall positive deviation with CO_2_, while for methane (CH_4_) they are comparable to Case 2. The anomaly observed in Case 4 was attributed to the assumptions made in respect of chemical potential. The profiles of gas concentration in the aqueous phase and their respective deviations were compared with the profiles of the parameters of nucleation to explain the influence of the gas dissolution parameters on the final output.

### 4.2. Rate of Nucleation

**Figure 7:**
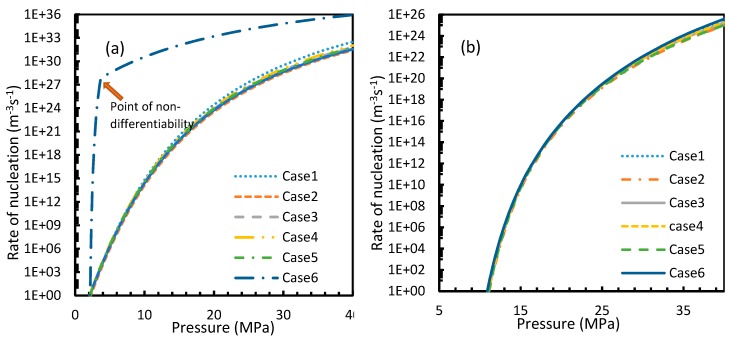
Profiles of nucleation rates of CO_2_ (**a**) and CH_4_ (**b**), under isothermal conditions with the reference temperature of 273.2 K. The profiles clearly show high rates of nucleation when CO_2_ was used as the guest gas compared to when CH_4_ was used. In addition, unlike the profiles of gas concentration in aqueous phase, the point of non-differentiability is shown only by Case 6.

**Figure 8:**
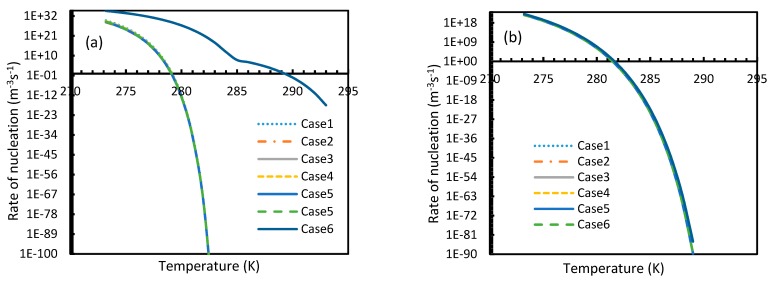
Profiles of nucleation rates of CO_2_ (**a**) and CH_4_ (**b**), under isobaric conditions with reference pressure 30 MPa. The profiles of all considered cases except Case 6 clearly show high depletions in CO_2_ nucleation rate values at high operational temperatures as compared to CH_4_, which raised a fundamental concern. In addition, the point of non-differentiability is shown only by Case 6, which was similar to Figure 7.

CO_2_ hydrate showed high rates of nucleation under isothermal conditions, where nucleation was observed to be initiated at pressures as low as 2.1 MPa, while the values of the nucleation rate of CH_4_ touched 10 m^−3^s^−1^ at pressures between 11–12 MPa, which was comparable to the value deduced by Kashchiev and Firoozabadi [10] (Figure 7). However, under isobaric conditions, all cases except for Case 6 showed early reductions in the rate of nucleation of CO_2_ at higher temperatures when compared to CH_4_ (Figure 8).

**Figure 9:**
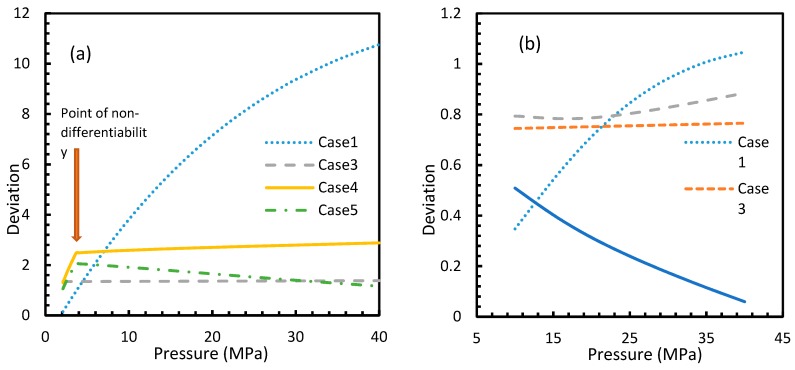
Deviation profiles for nucleation rates in Case 1 and Cases 3–5 of CO_2_ (**a**) and CH_4_ (**b**) from Case 2 under isothermal conditions with the reference temperature of 273.2 K. These profiles are observed to be the same as the profiles shown in dissolved concentration profiles.

**Figure 10:**
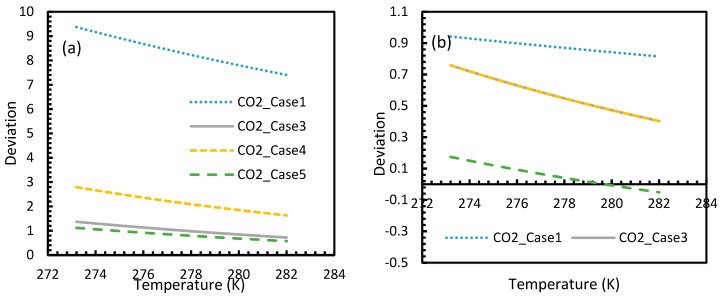
Profiles of nucleation rate deviations of CO_2_ (**a**) and CH_4_ (**b**) under isobaric conditions at a reference pressure 30 MPa, calculated through the considered cases apart from Case 6, from Case 2. The profiles are the same as the profiles shown in dissolved concentration profiles.

**Figure 11:**
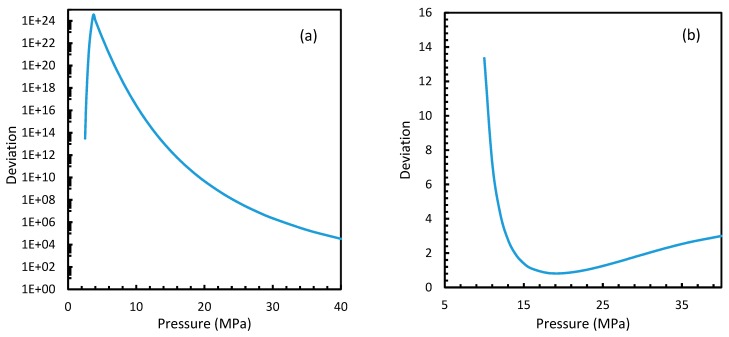
Profiles of deviations of nucleation rates of CO_2_ (**a**) and CH_4_ (**b**) under isobaric conditions with the reference temperature of 273.2 K, calculated through Case 6, from Case 2. The profiles show asymptotic curves, generally high deviations at lower pressure conditions that were dropped as the pressure increased. The deviations are immense for CO_2_ nucleation when compared to CH_4_. For CH_4_, high deviations were observed near at low pressure or at unfavorable hydrate conditions.

**Figure 12:**
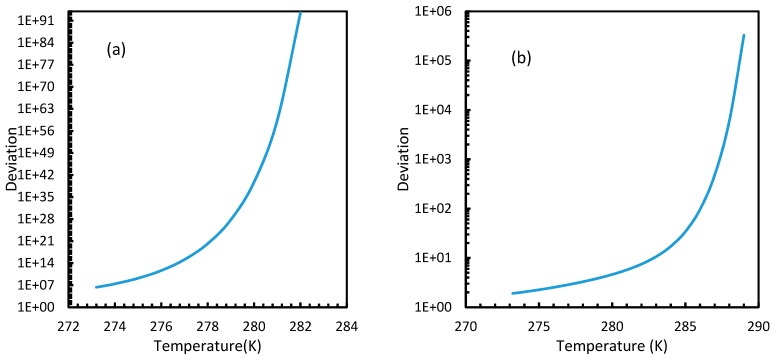
Profiles of deviations of nucleation rates of CO_2_ (**a**) and CH_4_ (**b**) under isobaric conditions at a reference pressure 30 MPa, calculated through Case 6, from Case 2. The profiles show exponential curves with less deviation at lower temperature and were increased as temperature increased. Relatively, the CO_2_ nucleation rate showed profound deviation from Case 2 values when compared to CH_4_.

From Equation (17), it was observed that the rate of nucleation was linearly related to the concentration of dissolved gas. Hence, the deviations shown in the profiles of rate of nucleation matched the deviations shown in the profiles of the dissolved gas concentration (Figure 5, Figure 6, Figure 9, and Figure 10), except for Case 6 for both CO_2_ and CH_4_ systems. The deviations shown by Case 6 were considerably higher, which suggested that the rate of nucleation equation was highly sensitive towards the driving force, whose equation was designed in terms of fugacity. At 5 MPa and 273.2 K, the rate of nucleation calculated for Case 6 was 3.28 × 10^28^ m^−3^ s, while for Case 2 it was 9.98 × 10^12^ m^−3^s (Figure 7a). For Case 6, the deviations were higher at lower pressures, and decrease with increasing pressure as well as increasing with temperature (Figure 11 and Figure 12). Hence, it was concluded that the deviations were lower at operational conditions favorable for hydration of high pressure and low temperature. From the values of the deviations presented in Figure 11 and Figure 12 of the rates of nucleation in Case 6, the sensitivity of hydrate nucleation to fugacity could be realized. However, the profiles for CH_4_ showed less deviation when compared to CO_2_.

The point of non-differentiability that was mentioned in Section 4.1 was observed only in Case 6 under isothermal and isobaric conditions, which was not in accordance with the concentration of dissolved gas profiles. This meant that the influence of dissolution was not sufficient to change the profiles of the rate of nucleation as much as the coefficient of fugacity. The main difference between CO_2_ and CH_4_ is the phase behavior under conditions of hydrate formation. Under the operational conditions, the compressibility of CH_4_ is negligible when compared to CO_2_, which makes CO_2_ a near real gas while CH_4_ is a near ideal gas.

**Figure 13:**
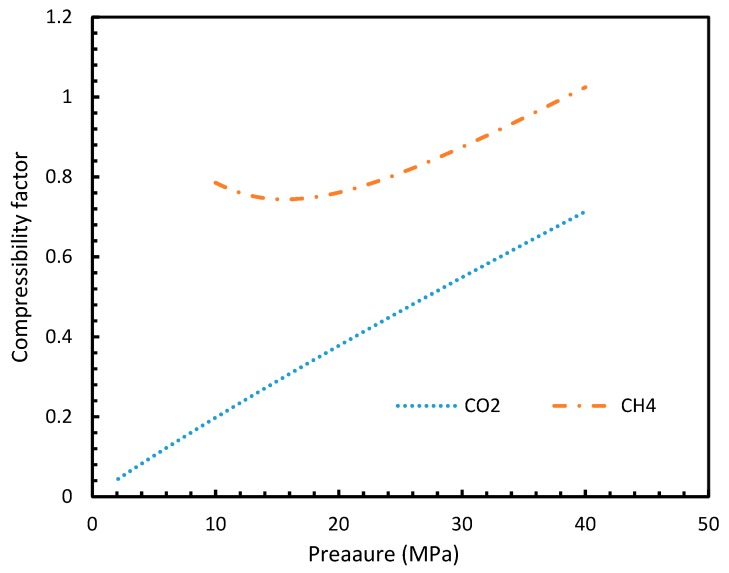
Compressibility factors of CO_2_ and CH_4_ under a range of operational pressures.

This was seen in Figure 13, where the compressibility factors of both CH_4_ and CO_2_ were plotted against operational temperature and pressure. This could be further explained in terms of the phase behavior of the guest gases under the operational conditions. The CO_2_ was able to change from the gaseous to the liquid phase and vice versa, under the hydrate formation conditions, while CH_4_ cannot, thus making CH_4_ a super-critical fluid, and hence a near ideal gas.

**Figure 14:**
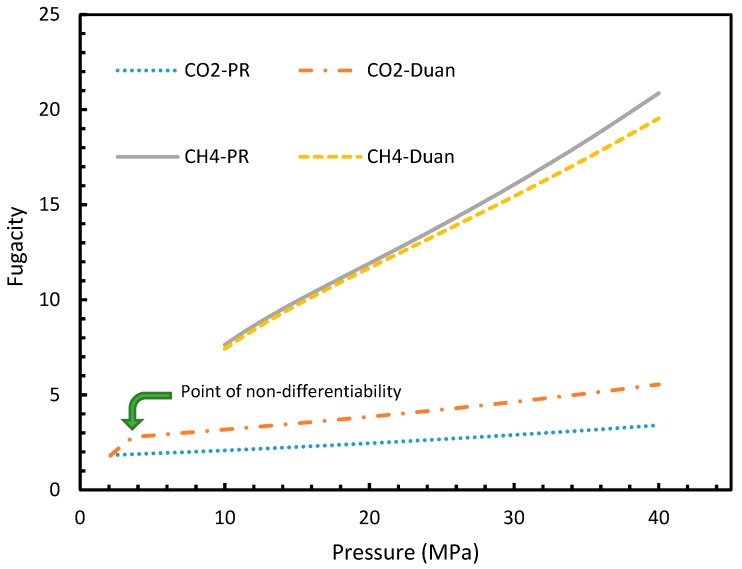
Comparison of Peng-Robinson (PR) and Duan fugacity in the case of CO_2_ and CH_4._

It is known that the conventional models for dissolution are empirical formulations, where the correlations for internal parameters are fabricated in such a way that final output, which is their combination, would result in a near practical value. Unlike the rest of the cases, which produced smooth profiles in their rates of nucleation against pressure and temperature, Case 6 produced non-differentiable profiles for its rates of nucleation for both isothermal and isobaric conditions. This explained the excess sensitivity of Kashchiev and Firoozabadi model’s nucleation towards the fugacity over extent of gas dissolution. In Figure 14, the fugacity calculated from PR and Duan models were compared where the point of non-differentiability was seen in the case of the Duan model, which had been causing the same in the rate of nucleation profiles. This point of non-differentiability was not observed in the case of CH_4_. However, there had been a considerable deviation at higher pressures.

**Figure 15:**
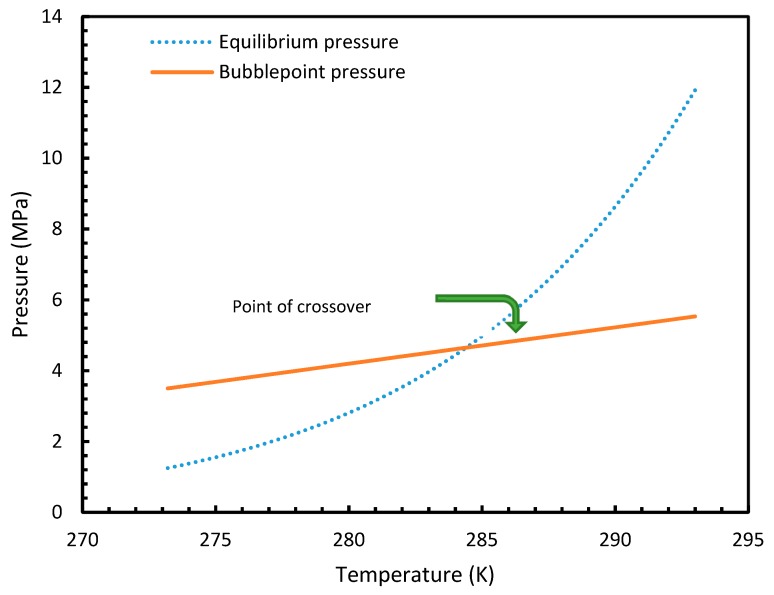
Profiles of equilibrium pressure and bubble point pressure of CO_2_ under the operational temperatures considered. The figure clearly shows the point of crossover at 285 K.

**Figure 16:**
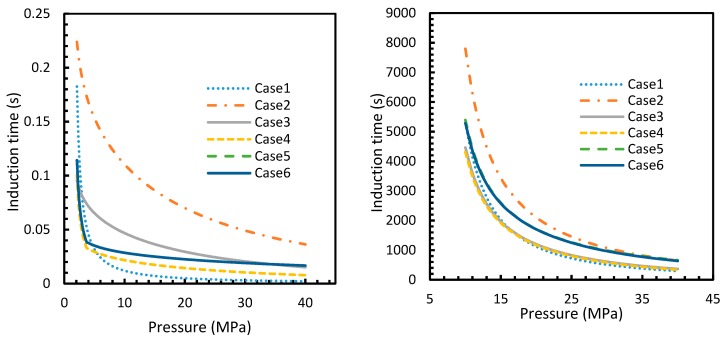
Profiles of CO_2_ (**a**) and CH_4_ (**b**) induction times, under isothermal conditions with the reference temperature of 273.2 K. The asymptotic profiles clearly show the relative rapidity of CO_2_ nucleation that were reflected in the values of theoretical induction time. The point of non-differentiability obtained in the CO_2_ induction time profiles, which is similar to the concentration of CO_2_ profiles shown in Figure 3a.

Similar to the situation in isothermal conditions, in isobaric conditions a point of non-differentiability was observed for Case 6. This resulted in higher rates of nucleation of CO_2_ at high temperatures compared to CH_4_. At 30 MPa and 285 K, Case 6 produced a rate of nucleation of CO_2_ of 3.67 × 10^7^, while for CH_4_ the value was 4.25 × 10^−23^ and values for the other cases were below 10^−100^ at 283 K for CO_2_. Despite CO_2_ being a significantly higher dissolvable gas than CH_4_, the plunge in CO_2_ consumption caused by these lowered nucleation rates can be experimentally observable when compared against CH_4_. This is because the amount of gas consumption due to the hydrate formation is profoundly higher when compared to the gas consumption contributed by dissolution. In the case of Case 6, the non-differentiability of nucleation rate profiles in isobaric conditions were not explained through fugacity, but from the crossover of bubble point pressure with the equilibrium pressure of CO_2_ is shown in Figure 15. Duan et al., in their solubility model, used various sets of fugacity correlations depending upon the experimental temperature [23]. This crossover triggered the change of correlation for equilibrium fugacity which affected the super saturation (Δμ) influencing the rate of nucleation profiles, which resulted in higher nucleation rates for CO_2_ at high temperatures. Studies such as Giavarini et al. (2007) [41] stressed the ease of CO_2_ hydrate formation compared to CH_4_ which were found to be supporting this observation superficially [41]. However, their observations were limited to low temperature conditions. To analyze it further, Figure 16 shows the hydrate/liquid/gas equilibrium pressures of CO_2_ and CH_4_ that have been derived from the model presented by Chapoy et al. (2014) [42]. According to this, CO_2_ is harder to form hydrates at higher temperatures when compared to CH_4_ due to the exponential raise in CO_2_ hydrate/liquid/gas equilibrium pressures. Along with practical observations, this statement was supported by other studies such as Daraboina et al. (2014) and Aresta et al., (2016) [43,44]. From Figure 10a, it is clearly seen that Case 6 suggested rates of nucleation trends that greatly deviate from the practical observations. It can be inferred that the semi-empirical Duan’s model failed to produce accurate fugacity values that could be applied for nucleation calculations, despite being applicable in wide ranges of pressure and temperatures for calculating gas dissolution, [22,23,24,25].

### 4.3. Induction Time

**Figure 17:**
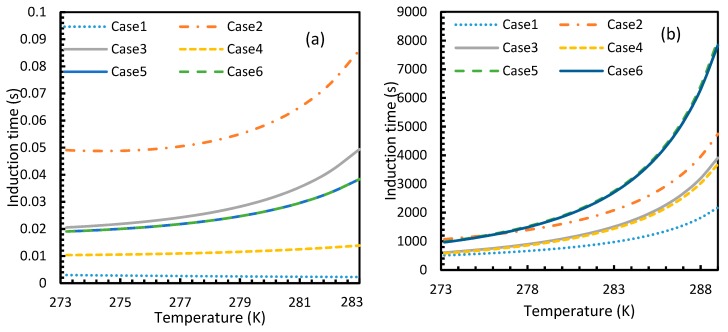
Profiles of theoretical of CO_2_ (**a**) and CH_4_ (**b**), under isobaric conditions with reference pressure 30 MPa. Despite the early depletion of CO_2_ hydrate nucleation at relatively lower temperatures in the cases, apart from Case 6, the theoretical induction time’s values are still considerably lower in case of CO_2_ than CH_4_, which can be seen.

The results from Equation (24) may not be reflected in experiments as the induction time was confined within the assumptions mentioned in Section 2.1. However, it is possible to use this theoretical induction time in calibrating ease of hydrate formation by comparing the performance of various pure guest gas systems. Figure 16 and Figure 17 explicitly show the enormous difference in the induction times of CO_2_ and CH_4_. These figures indicate the rapidity of CO_2_ hydrate formation, while suggesting a considerably longer induction time for CH_4_ hydrate.

**Figure 18:**
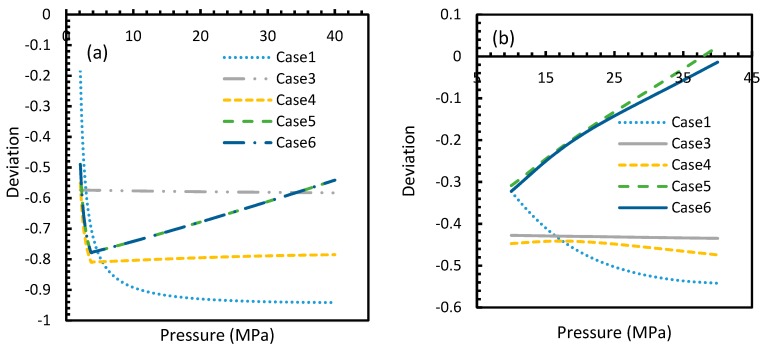
Profiles of deviations of theoretical induction times of CO_2_ (**a**) and CH_4_ (**b**) under various operational pressure conditions with the reference temperature of 273.2 K, calculated through the considered cases, apart from Case 6, from Case 2. The shapes of the profiles look like the inverted deviation profiles of nucleation rates and concentration of guest gas in aqueous phase only differing in values. Point of non-differentiability is pronounced by Cases 4, 5 and 6. In addition, in the profiles of Cases 5 and 6, a tendency of crossover into positive deviation can be observed.

**Figure 19:**
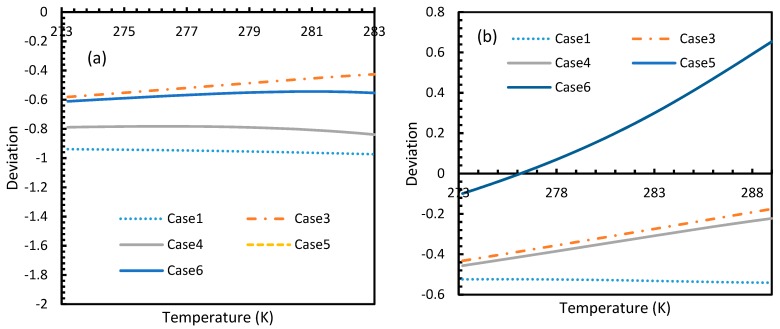
Profiles of deviations of theoretical induction times of CO_2_ (**a**) and CH_4_ (**b**) under isobaric conditions with reference pressure 30 MPa, calculated through the considered cases, apart from Case 6, from Case 2. Similar to Figure 17, the shapes of the profiles look like the inverted deviation profiles of nucleation rates and concentration of guest gas in aqueous phase only differing in quantitative perspective. The point of differentiability is pronounced by Cases 4, 5 and 6. A tendency of crossing over into positive deviation at higher temperatures can be observed in Case 5 and 6 when CO_2_ was used. However, a full crossover can be seen at approximately 277 K, when CH_4_ was used.

However, from Figure 18 and Figure 19, the deviation profiles of induction times for CH_4_ and CO_2_ hydrates did not show considerable divergence among the cases as much as the nucleation rate profiles. This could be attributed to the lack of exponentiality in Equation (14). Unlike the rate of nucleation, induction time was able to reflect phase change behavior through the point of non-differentiability in the profiles generated from Cases 4–6. Under isothermal conditions, the induction times for Cases 1 and 2 exhibited steeper profiles than the trends produced by Cases 5 and 6 after 3.2 MPa, which indicated the difficulty of hydrate formation with CO_2_ liquid. This was confirmed in Figure 18a,b, where the deviation values progressed from negative values to positive with pressure.

When it comes to a quantitative comparison of theoretical induction times, at 10 MPa and 273 K the value for CO_2_ was found to be 0.029 s while for CH_4_ it was over 5200 s. At 30 MPa and 285 K, CO_2_ induction time was calculated to be 0.051 s, while for CH_4_ it was over 3600 s. Experimentally, induction time could be experienced or observed and defined in a multitude of processes, as previously explained. The concept of induction time varies from researcher to researcher, since there is, as yet, no well-established technique for the detection of universal theoretical induction time.

Theoretically, induction time is the time taken for hydrate formation to become spontaneous under given operational conditions. The steady state hydrate formation rate after achieving spontaneity could vary depending upon physical conditions, such as pressure and temperature, and chemical conditions, such as the presence of guest gas and any promoters included in the system. This could potentially cause differences in induction times from one detection technique to another. For example, when considering the analogous technique used by Kashchiev and Firoozabadi [10] to derive the induction time equation, the measurement of the temporal evolution of the intensity of light passing through the hydrate solution merely indicates the percentage volume of hydrate crystals in the solution at any given point of time, the induction time varies with the experimental considerations upon the percentage occupancy of hydrate crystals in the solution at the induction time. This makes the induction time highly subjective towards the concerned researcher’s opinion. The resulted error will be magnified for the slower hydrate formation conditions or guest gases, which in our studies is CH_4_.

This is why Kashchiev’s induction time was not considered directly in this study, while it was used to derive our Equation (24) for theoretical induction time. From Figure 17 and Figure 19, similar to the deviations in concentration of dissolved gas profiles, the deviations in induction time profiles followed either decremental or asymptotic profiles with less magnitude, shown in all the cases apart from Case 1 at high temperature and low-pressure conditions for CO_2_. However, for methane, these values from Cases 5 and 6 showed an overall tendency to deviate positively from Case 2 at higher temperatures. From these observations, similar to the concentration of dissolved gas, hydrate formation conditions would require correlations with high accuracy to get relatively accurate results of induction time for CO_2_, while this observation was only restricted to isothermal conditions for CH_4_. These observations may change under high temperature and pressure conditions for both the guest gases, as the deviation profiles were not asymptotic.

An attempt was made to verify the validity of Equation (24) through experimental analysis. Despite the assumption of homogeneity in the model, to convert the induction time equation (Equation (24)) practicable, heterogeneity was introduced into it by adjusting the specific surface energy parameter (σ_ef_) as shown in Kashchiev and Firoozabadi [10]. Two hydrate formation experiments were conducted in the quiescent system, representing CO_2_ and CH_4_ hydrate formation in distilled water. An experimental pressure of 3.5 MPa was chosen for CO_2_ which had been 12 MPa for CH_4_ at 274.15 K. These operational conditions were chosen well above the equilibrium conditions and hydrate meta-stable state in order to provide suitable conditions for a progressive nucleation. This is supported by lesser observed induction times at operational conditions well above the hydrate equilibrium conditions [45,46]. Since this study was mainly focused on the primary nucleation, other physical interventions such as stirring were not included. When the temperature was decreased after the stabilization of experimental pressure and gas supply, an immediate gas consumption was observed with the temperature drop. This gas consumption was largely contributed by the contraction of gas in the system with the temperature drop, while a very small fraction was contributed by the excess gas dissolution into the water phase. From the experimental perspective, the induction time was considered to be the time taken for the system to show the first change in volumetric gas consumption after the consumption caused by temperature drop. For having less superficial energy barriers towards hydrate formation and highest thermal interaction with the cooling system, the most favorable regime of hydrate formation would be at the wall of the stainless-steel reactor [47,48].

**Figure 20:**
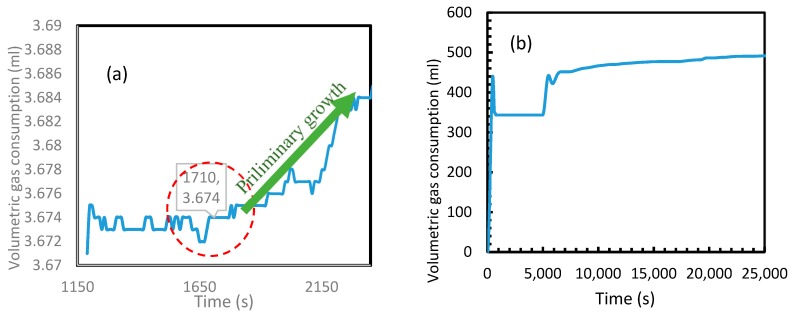
CH_4_ (**a**) and CO_2_ (**b**) volumetric gas consumption during the hydrate formation. Induction time was more visible in the case of methane than CO_2_ due to the exponential hydrate formation in the latter.

Figure 20 depicts the volumetric gas consumption of CH_4_ (a) and CO_2_ (b). In order to highlight the primary nucleation in the case of CH_4_, a period of 2000 s immediately after an active dissolution is shown in the Figure 20a. Even though the primary nucleation was observed to be heterogeneous, similar to the calculated induction times, a huge difference was found in between CO_2_ and CH_4_ hydrate formations. As seen in Figure 20b, in the case of CO_2_, for having exponential gas consumption, the consumption contributed by temperature drop and hydrate nucleation were indistinguishable. However, the induction time in case of CH_4_ was observed to be approximately 1700 s. In the case of CH_4_ gas consumption, the volumetric gain at 1700 s was relatively less than the gain observed later. For CH_4_ being a poorly soluble gas in water, gas hydrate formation always starts in the gas-liquid (g-l) interface at the wall and propagates into the interface before achieving the bulk nucleation and thereby forming hydrates in the bulk medium [47,49]. For having relatively less water molecules in the g-l interface along the wall against the water molecules in bulk, the volumetric gain observed during the nucleation in this medium was considerably less. To eliminate any confusion regarding the contribution of further gas consumption for volume gain after 1200 s, Figure 20a shows the steady state in volumetric CH_4_ consumption after an active dissolution before the preliminary growth after 1710 s. This observed induction time was further analyzed to assess the validity of the Equation (24), by calculating the water surface energy with the stainless-steel surface by substituting this experimental induction time into the equation. This water surface energy was used to calculate the water contact angle with stainless steel surface which was compared with the literature value. From the literature, it was understood that the contact angle and surface energy of any material with water are dependent upon the roughness of the material as well as its composition [50].

The studies indicate that the contact angle of water upon stainless steel can be in between 60° to 80° in the case of a smooth surface [50,51]. In the current study, assuming a relatively smooth surface, the contact angle of 66.2° was taken following the studies presented by Kalin and Polajnar (2014) [51]. Table 3 shows the contact angles calculated in this study among the six cases which were compared against the literature. It was seen that the Cases 5 and 6 produced contact angles with minimal error percentages with the literature value, while the rest deviated over 5%.

### 4.4. Hydrate Volume Fraction at Theoretical Induction Time

**Figure 21:**
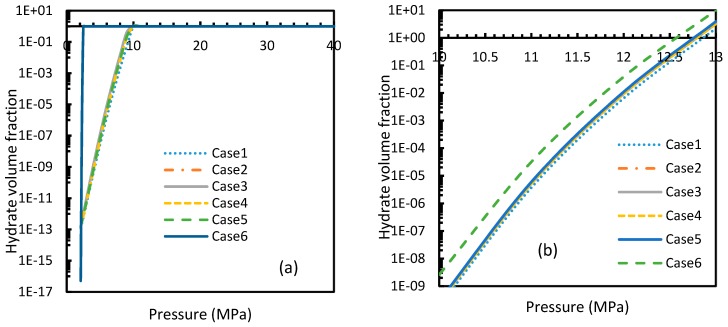
Profiles of hydrate volume fractions at theoretical induction time of CO_2_ (**a**) and CH_4_ (**b**) under isothermal conditions with the reference temperature of 273.2 K, calculated through the considered cases, apart from Case 6, from Case 2. It can be seen that at sufficient pressures, the hydrate formation becomes rapid enough to instantaneously shift to 100% conversion by the time of theoretical induction time. Additionally, a profound willingness of CO_2_ hydrate formation can be seen in the profile case 6.

Figure 21a shows the rapidity of CO_2_ hydrate nucleation at pressures as low as 9–10 MPa, where 100% conversion was achieved by the theoretical induction time. This observation was supported by the theoretical value of induction time calculated in Section 4.3, which showed an almost negligible induction time.

**Figure 22:**
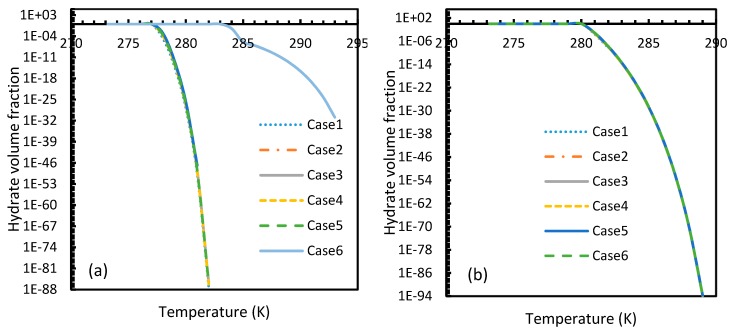
Profiles of hydrate volume fractions at theoretical induction time of CO_2_ (**a**) and CH_4_ (**b**) under isobaric conditions with reference pressure 30 MPa, calculated through the considered cases, apart from Case 6, from Case 2. The point of non-differentiability is found in case of Case 6 for CO_2_ hydrates, indicating relative favorability of CO_2_ for hydrate formation at high temperature, while the rest shows early depletion at relatively lower temperatures.

Even though CH_4_ hydrates needed a little increment of pressure to reach 100% conversion by the theoretical induction time, overall, they required higher pressure than CO_2_ (Figure 21). Among all the cases, the most rapid formation of both CO_2_ and CH_4_ hydrates occurred in Case 6. However, in this case a sharp rise in the profile of the CO_2_ hydrate volume fraction was observed at a lower operational pressure. Similar to the profiles of nucleation rates as mentioned in Section 4.2, volume fractions of CO_2_ showed an early depletion in their values at relatively lower temperatures to CH_4_ hydrates, while Case 6 showed otherwise (Figure 22), limiting the applicability of Duan’s fugacity.

From these results, it was concluded that the volume fraction of hydrates at the end of the theoretical induction time were not constant but dynamic. Hence, the observed induction time through temporal evolution of intensity of projected light might not be the same as the time hydrate formation become spontaneous. The reason could be ascribed to variable critical radius at various operational conditions. Due to mutual cancellation of deviations contributed by various internal parameters, the critical radius did not show any deviations among the considered cases.

## 5. Future Perspectives

In order to assess the hydrate formation process without having to conduct the experiments, a model has to be developed that connects theory with the practical observations. Including our experimental observations, various results showed the hydrate formation occurs on a substrate posing less surface energy and progresses thereafter. The process is temporarily decelerated or stopped when the heat in the regime increases. Hence, hydrate formation should not be seen as a mere crystallization process but a combination of gas dissolution, heat transfer and crystallization together. In the systems having electrolytes, this assessment becomes more complex for having mass transfer coming into the picture. In the current study, we have started at the theoretical concepts of hydrate nucleation and tried to check its sensitivity towards generic and empirical/semi-empirical models. For having no prior hydrate formation, this evaluation was rather simpler than the cases having the presence of hydrates. This is because we did not require three phase (gas-liquid-hydrate) equilibrium models and also thermal fluctuations were neglected. From our analysis, Case 5 had more plausibility towards practical applications in the field of progressive nucleation among the other cases. However, the hydrate formation as a whole being more complicated than just a progressive nucleation, this study gives a scope to improve the existing model by involving other phenomena co-occurring along with hydrate formation. Through this study, we have tried to give a generic and theoretical definition for induction time and checked its validity indirectly by calculating the water-stainless steel contact angle from it. However, this induction time may not ensure an exponential hydrate formation. Therefore, hydrate nucleation is considered to be stochastic. This stochastic nature increases with the inefficiency of the hydrate former (guest gas), which has been seen experimentally in the case of CH_4_ hydrate formation. However, there is still scope to improve mathematical techniques to measure the induction time for efficient hydrate formers.

## 6. Conclusions

Hydrate parameters such as rate of nucleation, induction time and hydrate volume fraction at the theoretical induction time were calculated under pressure and temperature conditions favorable for hydrate formation in CO_2_ and CH_4_ guest gas systems. Various existing mathematical models were used to calculate the dissolved gas concentration and fugacity of CO_2_ and CH_4_. The analysis was divided into six cases with each case altering one correlation at a time to calculate an intermediate parameter that was further used to calculate the rate of nucleation. From this comparative study, we concluded that the application of empirical/semi-empirical models were to be done carefully as the correlations for their intermediate parameters might not be accurate, despite fulfilling their purpose overall. Between the guest gases, CO_2_ showed higher deviations than CH_4_ and hence higher vulnerability towards inaccuracies posed by various correlations. From the values and profiles of the parameters considered against various operational temperatures and pressures, CO_2_ was observed to be more favorable for hydrate formation than CH_4_ by a considerable margin. Equations for theoretical induction time and the volume fraction of hydrates at that time were derived from existing models. Furthermore, the values derived by using our theoretical induction time equation were compared against the experimental results indirectly. Amongst all the cases, Case 5 and 6 were proven to be more effective in calculating the induction time. When considered both induction time and rate of nucleation aspect, only Case 5 showed the best practicability among the rest of the cases. This suggests the need to calculate fugacity from both theoretical and semi-empirical methods to be able to analyze the process of nucleation through the process proposed by Kashchiev and Firoozabadi.

## Figures and Tables

**Table 1:** Parameters of dimensionless Henry’s constant (k_H_) to be substituted in Equation (3) [29].

	A_H_	B_H_	C_H_
CO_2_	−9.4234	4.0087	10.3199
CH_4_	−11.0094	48362	12.5220

**Table 2:** Parameters of Equations (6) and (7) (Duan et al. [22] for CH_4_ and Duan et al. for CO_2_ [22,24].

	CO_2_		CH_4_
	1	2	
C_1_	1.0	−7.1734882 × 10^−1^	8.72553928 × 10^−2^
C_2_	4.7586835 × 10^−3^	1.5985379 × 10^−4^	−7.52599476 × 10^−1^
C_3_	−3.3569963 × 10^−6^	−4.9286471 × 10^−7^	3.75419887 × 10^−1^
C_4_	0.0	0.0	1.07291342 × 10^−2^
C_5_	−1.3179396	0.0	5.49626360 × 10^−3^
C_6_	−3.8389101 × 10^−6^	−2.7855285 × 10^−7^	−1.84772802 × 10^−2^
C_7_	0.0	1.1877015 × 10^−9^	3.18993183 × 10^−4^
C_8_	2.2815104 × 10^−3^	0.0	2.11079375 × 10^−4^
C_9_	0.0	0.0	2.01682801 × 10^−5^
C_10_	0.0	0.0	−1.65606189 × 10^−5^
C_11_	0.0	0.0	1.19614546 × 10^−4^
C_12_	0.0	−96.539512	−1.08087289 × 10^−4^
C_13_	0.0	4.4774938 × 10^−1^	4.48262295 × 10^−2^
C_14_	0.0	101.81078	7.53970000 × 10^−1^
C_15_	0.0	5.3783879 × 10^−6^	7.71670000 × 10^−2^

1: P < P_b_ (P_b_ is the bubble point pressure of CO_2_); 2: P > P_b_.

**Table 3:** Contact angles calculated from the considered cases.

S.No	Cases	Contact Angle	Percentage Deviation
1	Case 1	74.06	11.88
2	Case 2	59.82	−9.64
3	Case 3	77.29	16.75
4	Case 4	78.61	18.74
5	Case 5	68.70	3.77
6	Case 6	69.16	4.47

## References

[1] He Z., Linga P., Jiang J. (2017). What are the key factors governing the CO_2_ hydrates?. Energy Fuels.

[2] Eslamimanesh A., Mohammadi A.H., Richon D., Naidoo P., Ramjugernath D. (2012). Application of gas hydrate formation in separation processes: A review of experimental studies. J. Chem. Thermodyn..

[3] Ribeiro C.P., Lage P.L. (2008). Modelling of hydrate formation kinetics: State-of-the-art and future directions. Chem. Eng. Sci..

[4] Linga P., Clarke M.A. (2016). A review of reactor designs and materials employed for increasing the rate of gas hydrate formation. Energy Fuels.

[5] Englezos P., Kalogerakis N., Dholabhai P.D., Bishnoi P.R. (1987). Kinetics of gas hydrate formation from mixtures of methane and ethane. Chem. Eng. Sci..

[6] Skovborg P., Ng H.J., Rasmussen P., Mohn U. (1993). Measurement of induction times for the formation of methane and ethane gas hydrates. Chem. Eng. Sci..

[7] Natarajan V., Bishnoi P.R., Kalogerakis N. (1994). Induction phenomena in gas hydrate nucleation. Chem. Eng. Sci..

[8] Herri J.M., Gruy F., Pic J.S., Cournil M., Cingotti B., Sinquin A. (1999). Experiments on the quantity of gases absorbed by water, at different temperatures, and under different pressures. Chem. Eng. Sci..

[9] Kashchiev D., Firoozabadi A. (2002). Driving force for crystallization of gas hydrates. J. Cryst. Growth.

[10] Kashchiev D., Firoozabadi A. (2002). Nucleation of gas hydrates. J. Cryst. Growth.

[11] Kashchiev D., Firoozabadi A. (2003). Induction time in crystallization of gas hydrates. J. Cryst. Growth.

[12] Anklam M.R., Firoozabadi A. (2004). Driving Force and Composition for Multicomponent Gas Hydrate Nucleation from Supersaturated Aqueous Solutions. J. Chem. Phys..

[13] Sloan E.D., Koh C. (2007). Clathrate Hydrates of Natural Gases.

[14] Kazemeini M., Freidoonian F., Fattahi M. (2012). Developing a Mathematical Model for Hydrate Formation in a Spray Batch Reactor. Adv. Mater. Phys. Chem..

[15] Kashchiev D. (2000). Nucleation.

[16] Henry W. (1803). Experiments on the Quantity of Gases Absorbed by Water, at Different Temperatures, and under Different Pressures. Philos. Trans. R. Soc. Lond..

[17] Raoult F.M. (1887). Loi générale des tensions de vapeur des dissolvants. C. R. Hebd. Séances Acad. Sci..

[18] King M.B., Mubarak A., Kim J.D., Bott T.R. (1992). The mutual solubilities of water with supercritical and liquid carbon dioxides. J. Supercrit. Fluids.

[19] Michels A., Gerver J., Bijl A. (1936). The influence of pressure on the solubility of gases. Physica.

[20] Wiebe R., Gaddy V.L. (1939). The solubility in water of carbon dioxide at 50, 75 and 100, at pressures to 700 atmospheres. J. Am. Chem. Soc..

[21] Blanco C.L.H., Smith N.O. (1978). The high-pressure volubility of methane in aqueous calcium chloride and aqueous tetraethylammonium bromide. Partial molar properties of dissolved methane and nitrogen in relation to water structure. J. Phys. Chem..

[22] Duan Z., Møller N., Greenberg J., Weare J.H. (1992). An equation of state for the CH_4_-CO_2_-H_2_O system: II. Mixtures from 50 to 1000 °C and 0 to 1000 bar. Geochim. Cosmochim. Acta.

[23] Duan Z., Sun R. (2003). An improved model calculating CO_2_ solubility in pure water and aqueous NaCl solutions from 273 to 533 K and from 0 to 2000 bar. Chem. Geol..

[24] Duan Z., Mao S. (2006). A thermodynamic model for calculating methane solubility, density and gas phase composition of methane-bearing aqueous fluids from 273 to 523 K and from 1 to 2000 bar. Geochim. Cosmochim. Acta.

[25] Duan Z., Sun R., Zhu C., Chou I.M. (2006). An improved model for the calculation of CO_2_ solubility in aqueous solutions containing Na^+^, K^+^, Ca^2+^, Mg^2+^, Cl^−^, and SO_4_^2−^. Mar. Chem..

[26] Park K.N., Hong S.Y., Lee J.W., Kang K.C., Lee Y.C., Ha M.G., Lee J.D. (2011). A new apparatus for seawater desalination by gas hydrate process and removal characteristics of dissolved minerals (Na^+^, Mg^2+^, Ca^2+^, K^+^, B^3+^). Desalination.

[27] Ota M., Morohashi K., Abe Y., Watanabe M., Smith R.L., Inomata H. (2005). Replacement of CH_4_ in the hydrate by use of liquid CO_2_. Energy Convers. Manag..

[28] Kim N.J., Lee J.H., Cho Y.S., Chun W. (2010). Formation enhancement of methane hydrate for natural gas transport and storage. Energy.

[29] Anderson R., Llamedo M., Tohidi B., Burgass R.W. (2003). Experimental measurement of methane and carbon dioxide clathrate hydrate equilibria in mesoporous silica. J. Phys. Chem. B.

[30] Bergeron S., Servio P. (2009). CO_2_ and CH_4_ mole fraction measurements during hydrate growth in a semi-batch stirred tank reactor and its significance to kinetic modeling. Fluid Phase Equilibria.

[31] Hashemi S., Macchi A., Bergeron S., Servio P. (2006). Prediction of methane and carbon dioxide solubility in water in the presence of hydrate. Fluid Phase Equilibria.

[32] Lang F. (2016). Liquid Phase Characterization of Multicomponent Gas Hydrate Systems. Ph.D. Thesis.

[33] Sander R. (2015). Compilation of Henry’s law constants (version 4.0) for water as solvent. Atmos. Chem. Phys..

[34] Japas M.L., Sengers J.M.H. (1989). Gas solubility and Henry’s law near the solvent’s critical point. AIChE J..

[35] Harvey A.H. (1996). Semiempirical correlation for Henry’s Constants over Large Temperature Ranges. AIChE J..

[36] Diamond L.W., Akinfiev N.N. (2003). Solubility of CO_2_ in water from −1.5 to 100 °C and from 0.1 to 100 MPa: Evaluation of literature data and thermodynamic modelling. Fluid Phase Equilibria.

[37] Renon H., Prausnitz J.M. (1968). Local compositions in thermodynamic excess functions for liquid mixtures. AIChE J..

[38] Vera J.H., Sayegh S.G., Ratcliff G.A. (1977). A quasi lattice-local composition model for the excess Gibbs free energy of liquid mixtures. Fluid Phase Equilibria.

[39] Islam A.W., Carlson E.S. (2012). Activity Coefficient Models for Calculations of Supercritical CO_2_ and H_2_O at High Temperatures and Pressures. GRC Trans..

[40] Denbigh K.G. (1981). The Principles of Chemical Equilibrium: With Applications in Chemistry and Chemical Engineering.

[41] Giavarini C., Maccioni F., Politi M., Santarelli M.L. (2007). CO_2_ Hydrate: Formation and Dissociation Compared to Methane Hydrate. Energy Fuels.

[42] Chapoy A., Burgass R., Tohidi B., Alsiyabi I. (2014). Hydrate and phase behavior modeling in CO_2_-rich pipelines. J. Chem. Eng. Data.

[43] Aresta M., Dibenedetto A., Quaranta E. (2016). Thermodynamics and Applications of CO_2_ Hydrates. Reaction Mechanisms in Carbon Dioxide Conversion.

[44] Herslund P.J., Daraboina N., Thomsen K., Abildskov J. Modelling and Measuring Hydrate Promotion for CO_2_ Capture. Proceedings of the 8th International Conference on Gas Hydrates (ICGH8-2014).

[45] Vysniauskas A., Bishnoi P.R. (1983). A kinetic study of methane hydrate formation. Chem. Eng. Sci..

[46] Parent J.S., Bishnoi P.R. (1996). Investigations into the nucleation behaviour of methane gas hydrates. Chem. Eng. Commun..

[47] Long J.P., Sloan E.D. (1996). Hydrates in the ocean and evidence for the location of hydrate formation. Int. J. Thermophys..

[48] Takeya S., Hori A., Hondoh T., Uchida T. (2000). Freezing-memory effect of water on nucleation of CO_2_ hydrate crystals. J. Phys. Chem. B.

[49] Gayet P., Dicharry C., Marion G., Graciaa A., Lachaise J., Nesterov A. (2005). Experimental determination of methane hydrate dissociation curve up to 55 MPa by using a small amount of surfactant as hydrate promoter. Chem. Eng. Sci..

[50] Prajitno D.H., Maulana A., Syarif D.G. (2016). Effect of surface roughness on contact angle measurement of nanofluid on surface of stainless steel 304 by sessile drop method. J. Phys. Conf. Ser..

[51] Kalin M., Polajnar M. (2014). The wetting of steel, DLC coatings, ceramics and polymers with oils and water: The importance and correlations of surface energy, surface tension, contact angle and spreading. Appl. Surf. Sci..

